# Retinoprotective Effects of Abscisic Acid in Ischemic Retinopathy Mouse Model

**DOI:** 10.3390/antiox14091133

**Published:** 2025-09-19

**Authors:** Inez Bosnyak, Agnes Nagy, Dorottya Molitor, Balazs Meresz, Edina Szabo, Dora Reglodi, Tamas Atlasz, Alexandra Vaczy

**Affiliations:** 1 Department of Anatomy, HUN-REN-PTE PACAP Research Team, Medical School, University of Pecs, 7624 Pecs, Hungary; bosnyak.inez@aok.pte.hu (I.B.); molitor.dorottya@edu.pte.hu (D.M.); meresz.balazs@edu.pte.hu (B.M.); edina.szabo@aok.pte.hu (E.S.); dora.reglodi@aok.pte.hu (D.R.); alexandra.vaczy@aok.pte.hu (A.V.); 2 Center for Health Technology Assessment and Pharmacoeconomic Research, Faculty of Pharmacy, University of Pecs, 7624 Pecs, Hungary; nagy.agnes5@pte.hu; 3 Department of Sports Biology and Kinesiology, Faculty of Sciences, University of Pecs, 7624 Pecs, Hungary

**Keywords:** retina, hypoxia, abscisic acid, eye drops, protection

## Abstract

The prevalence of hypoxia-caused eye diseases is increasing, but effective, non-invasive treatment options are not available. Abscisic acid (ABA) is a plant hormone with anti-inflammatory and antioxidant effects. ABA is also present in various mammalian tissues and plays an important role in metabolic processes. Therefore, we aimed to investigate the potential protective role of ABA eye drops in ischemic retinopathy. Retinal ischemia was induced by permanent unilateral common carotid artery occlusion (UCCAO) in mice. Half of the animals received ABA eye drops two times a day for two weeks. Optical coherence tomography (OCT) was used to follow the changes in retinal thickness. Moreover, immunohistochemistry and molecular biology methods were used to explore the morphological changes and the underlying mechanisms, respectively. Based on OCT measurements, ischemic retinopathy was successfully developed. The decrease in the thickness of numerous retinal layers could be prevented with ABA eye drops. The ganglion cell number decreased significantly after UCCAO in the central and peripheral regions of the retina. ABA treatment could moderate the damage in both regions. Furthermore, our apoptosis array results suggest that ABA regulates the apoptotic pathways under hypoxic conditions. In conclusion, ABA eye drops may represent a new potential therapeutic option for the treatment of ischemic retinopathy.

## 1. Introduction

The leading causes of blindness include cataracts, glaucoma, uncorrected refractive error, age-related macular degeneration and diabetic retinopathy in people aged 50 and older [1]. In addition, retinopathy of prematurity commonly leads to visual impairment in infants also affecting the visual acuity in childhood and adulthood [2,3]. Although the prevalence of these diseases is increasing, their treatment remained a significant challenge. The management of diabetic retinopathy and retinopathy of prematurity includes intravitreal anti–vascular endothelial growth factor (anti-VEGF) therapy, laser photocoagulation, corticosteroids, and surgical procedures, whereas glaucoma treatment primarily targets reduction in intraocular pressure [2,4,5,6,7,8]. The exact pathomechanism of these conditions is not fully understood, but it is generally accepted that retinal hypoxia plays a key role in their development. Furthermore, morphological and functional studies indicate that inflammatory mediators and oxidative stress also contribute to the induction and progression of these pathologies [9,10,11,12,13,14]. Reactive oxygen species are produced by the family of NADPH oxidase enzymes in various pathways, including the mitochondrial electron transport chain. These enzymes are involved in pathological angiogenesis, which has a key role in the induction and progression of diabetic retinopathy and of retinopathy of prematurity [15]. It has been recently reviewed that antioxidant agents can moderate the functional loss in rodent models of diabetic retinopathy. Electroretinography measurements showed that antioxidant supplementation decreased the change in the amplitude of a- and b- waves after induction of diabetes [13].

Abscisic acid (ABA) is a sesquiterpenoid phytohormone regulating numerous physiological processes, including seed dormancy and germination. It is considered as a highly conserved universal stress signaling molecule activated by a wide range of endogenous or exogenous stimuli [16,17]. In 1986, ABA was described in the mammalian brain and, since then, many articles have been published on its functions in various mammalian tissues [17,18]. Notably, ABA enhances glucose uptake and increases energy production in adipose tissue and skeletal muscle via an insulin-independent pathway. Therefore, exogenous ABA supplements from fruits and vegetables are promising to improve glucose tolerance in prediabetic and diabetic patients. Furthermore, plasma ABA level increases following glucose intake, indicating endogenous ABA production in animals [17,19]. Beyond metabolism, ABA has gained attention for its potential neuroprotective effects. In rodent models, ABA has been shown to reduce locomotor hyperactivity, modulate social interaction in attention-deficit/hyperactivity disorder (ADHD), decrease motor, cognitive, and affective deficits in cerebellar ataxia, and ameliorate cognitive dysfunction associated with diabetes or essential tremor [20,21,22,23]. The positive effects of ABA on learning and memory, as well as other neurocognitive disorders, have been extensively reviewed [24,25,26,27].

Based on the literature, ABA can have a protective effect in many different diseases, largely due to its anti-inflammatory and antioxidant effects. It modulates the cytokine and chemokine production, macrophage infiltration, microglia activation and the nuclear expression of nuclear factor erythroid 2-related factor 2 (NRF2), among others [26,28,29,30,31,32,33]. ABA enhances the effectiveness of antioxidant defense systems through the enzymes glutathione reductase, superoxide dismutase, glutathione-S-transferase and catalase [34]. These effects suggest that ABA may also be protective in other, less studied organs, such as the eye.

It has been shown that ABA has no cytotoxic effect on cultured retinal endothelial cells. In the retina of newborn mice, it acted as an anti-angiogenic agent similar to the commonly used anti-VEGF [35]. In this study, ABA inhibited key steps of angiogenesis, including endothelial cell differentiation, migration, and proliferation. It also reduced vascular density, decreased endothelial tip cell numbers, and influenced macrophage polarization. Moreover, these processes occurred without increasing retinal stress. However, these effects were achieved via intravitreal injection, an invasive route of administration [35].

The present study was, therefore, designed to investigate whether ABA, delivered non-invasively as eye drops, can protect the retina in a mouse model of ischemic retinopathy.

## 2. Materials and Methods

### 2.1. Animals and Experimental Design

The study was carried out on 47 adult male CD1-IGS mice, 4–5 months old. They were maintained under controlled conditions, exposed to a 12 h light/12 h dark cycle, and provided food and water ad libitum. All procedures were in accordance with the institutional guidelines of the Animal Research Review Committee of the University of Pecs, Hungary (permission no. BA02/2000-02/2022), and conformed to the standards set forth by the European Communities Council Directive (86/609/EEC) and the ARVO Statement for the Use of Animals in Ophthalmic and Vision Research. After undergoing invasive or non-invasive examinations, mice were carefully monitored until they woke up. Animals were randomly divided into four experimental groups: control animals (*n* = 16) receiving vehicle (left eyes) or ABA (right eyes) treatment, a group subjected to permanent right-sided unilateral common carotid artery occlusion (UCCAO; *n* = 15), and a UCCAO group treated with ABA (*n* = 16).

### 2.2. Unilateral Common Carotid Artery Occlusion (UCCAO)

Retinal ischemia was induced by permanent UCCAO as described previously [36]. Surgical interventions were carried out under general anesthesia induced by intraperitoneal injection of xylazine (10 mg/kg; Sedaxylan, Dechra, Amsterdam, The Netherlands) and ketamine (90 mg/kg; Calypsol, Richter Gedeon, Budapest, Hungary). The cervical region was disinfected using Braunol antiseptic solution (B. Braun Medical AG, Sempach, Switzerland), and a cervical midline incision was made to expose the surgical field. The salivary glands and surrounding neck muscles were gently retracted to visualize the common carotid artery. Then the vagus nerve was separated from the artery to avoid damaging the nerve. In the UCCAO groups, the right common carotid artery was permanently occluded by placing two non-absorbable 3-0 sutures and the vessel was cut between the ligatures to avoid reperfusion. This method induces moderate hypoxia in the ipsilateral retina [36].

### 2.3. Topical Administration of ABA

Following UCCAO surgeries, the mice received topical ocular treatment twice daily for 14 consecutive days. One drop of either vehicle or ABA solution was administered to each eye at each time point. The ABA eye drops formulation (0.5 µg/drop) was prepared by initially dissolving 2 mg of abscisic acid (Sigma-Aldrich, Budapest, Hungary) in dimethyl sulfoxide (DMSO) (Sigma-Aldrich, Budapest, Hungary), followed by stepwise dilution with Systane^®^ lubricant eye drops (Alcon Kft., Budapest, Hungary) to achieve a final ABA concentration of 50 µM. The vehicle solution contained the same final concentration of DMSO in Systane without ABA.

### 2.4. Optical Coherence Tomography (OCT)

In vivo imaging of retinal morphology was conducted using spectral domain optical coherence tomography (SD-OCT) with a system from Leica Microsystem (Bioptigen, Morrisville, NC, USA). OCT measurements were performed before the surgeries (day 0) and 14 days later using a lens designed for mouse eyes, capturing rectangular scan areas of 1.8 mm × 1.8 mm. Each scan series consisted of 1000 A-scans per B-scan, with 100 B-scans per volume, and three frames were recorded per B-scan for averaging purposes. The mice were anesthetized via intraperitoneal injection of a ketamine (Calypsol, Richter Gedeon, Budapest, Hungary) and xylazine (Sedaxylan, Dechra, Amsterdam, The Netherlands) mixture to ensure immobilization during imaging. Pupil dilation was achieved using 0.01% atropine eye drops, and artificial tears (Systane, Alcon, Budapest, Hungary) were applied throughout the procedure to maintain corneal integrity and optimize imaging quality. UCCAO—Systane (*n* = 7) and UCCAO—ABA (*n* = 8) groups were included in this analysis and layers were compared to their initial values to avoid inter-individual variability. The thickness of the layers was quantified using the Bioptigen InVivoVue Diver software’s (version 3.3.7, Bioptigen Inc., Leica Microsystem, Durham, NC, USA) auto-segmentation function to ensure consistent delineation of the layer boundaries.

### 2.5. Photoreceptor Labeling in Retinal Cross Section

Fourteen days following surgical interventions, mice (*n* = 3 per group) were deeply anesthetized using isoflurane, after which euthanasia was performed via cervical dislocation. The eyes were gently enucleated by dissecting through the eyelids and severing the optic nerve. For immunofluorescence labeling, eyecups were carefully excised in ice-cold 0.1 M phosphate-buffered saline (PBS), then immersion-fixed in freshly prepared 4% paraformaldehyde (PFA) dissolved in 0.1 M PBS for 1 h at room temperature. Following fixation, tissues were rinsed thoroughly in 0.1 M PBS for an additional hour to remove excess fixative. After washing, the eyecups underwent sequential cryoprotection in 10%, 20%, and 30% sucrose solutions. Samples were then embedded in Tissue Freezing Medium (Mount Waverley, Australia) and rapidly frozen. Cryosections of 15 μm thickness were obtained from central retinal regions (within 2 mm from the optic nerve head) using a Leica CM1950 cryostat (BioMarker, Budapest, Hungary) and mounted on gelatin-coated microscope slides for subsequent immunolabeling. Before applying the primary antibodies, the sections were first rehydrated in 0.1 M PBS. They were then incubated for 2 h at room temperature in a blocking mixture composed of 5% normal donkey serum, 3% bovine serum albumin, and 0.3% Triton X-100 in PBS, to minimize unspecific antibody binding. Primary antibody incubation was performed overnight at 4 °C in a humidified chamber. Sections were incubated with (I) mouse anti-rhodopsin (1:500, MABN15, Merck Life Science Kft., Budapest, Hungary) and (II) rabbit anti-arrestin (1:250, ab15282, Merck Life Science Kft., Budapest, Hungary), both diluted in antibody diluent. The next day, following thorough washes in PBS secondary antibodies were applied for 2 h at room temperature: (I) Alexa Fluor 594-conjugated donkey anti-mouse IgG (715-585-150, Jackson ImmunoResearch, Cambridgeshire, UK) and (II) Alexa Fluor 488-conjugated donkey anti-rabbit IgG (711-545-152; Jackson ImmunoResearch, Cambridgeshire, UK), each diluted 1:1000 in PBS. After final PBS washes (1 h total), sections were mounted with Fluoroshield mounting medium (Sigma-Aldrich, Budapest, Hungary). For negative controls, the primary antibodies were omitted, which resulted in the absence of specific signal. Images were captured using a Nikon Eclipse 80i fluorescence microscope (Melville, NY, USA). Final image processing (contrast adjustment, alignment, and labeling) was carried out using Adobe Photoshop CS6 (Adobe Systems Inc., San Jose, CA, USA), without applying any modifications beyond linear contrast adjustment and layout optimization.

### 2.6. Detection of Retinal Ganglion Cells and Glial Activation by Whole-Mount Immunolabeling

Fourteen days following surgical interventions, mice (*n* = 3–5 per group) were deeply anesthetized, and eyes were isolated as described in the previous section. After extraction, the eyeballs were placed in 0.1 M PBS, and a small incision was made at the corneal limbus to facilitate fixation. Fixation was carried out in PFA prepared in 0.1 M PBS for 1 h at room temperature. For whole-mount preparations, the cornea and lens were carefully removed, and the retinas were isolated intact. Tissues were rinsed six times for 5 min each in 0.1 M PBS. Samples were then blocked for 1 h at room temperature in a solution containing 3% BSA, 4% NDS, and 0.5% Triton X-100 diluted in PBS. Retinas were divided into two sets for immunolabeling with either brain-specific homeobox/POU domain protein 3A (Brn3a), a specific marker of retinal ganglion cells, or glial fibrillary acidic protein (GFAP), which is overexpressed in activated glial cells. The first set was incubated overnight at 4 °C with a rabbit monoclonal anti-Brn3a primary antibody (1:50, ab245230 Abcam, Cambridge, UK), while the second set was incubated under identical conditions with a rabbit anti-GFAP primary antibody (1:250, G9269, Sigma-Aldrich, St. Louis, MO, USA), both diluted in the blocking buffer. Following primary antibody incubation, samples were washed six times in PBS (5 min each), then incubated with Alexa Fluor 594-conjugated donkey anti-rabbit IgG secondary antibody (1:800, 711-585-152, Jackson ImmunoResearch, Cambridgeshire, UK) for 1 h at room temperature. After staining, four radial incisions were made to flatten the retinas into a flower-like shape. Each retina was mounted onto a glass slide using Fluoroshield mounting medium (Sigma-Aldrich, Budapest, Hungary). To assess nonspecific binding, control sections were incubated without primary antibodies and showed no specific fluorescence signal. Fluorescent imaging was performed using a Nikon Eclipse 80i microscope (Melville, NY, USA). Images were taken from both central (*n* = 8–15 different regions from the 4–5 animals) and peripheral (*n* = 10–18 different regions from the 4–5 animals) retinal areas. Quantification of Brn3a-positive retinal ganglion cells was performed with an automated cell counting method using ImageJ software (version 1.54g). GFAP staining was used only for representative purposes to visualize changes in glial morphology. To ensure consistent image quality, all micrographs were acquired under identical conditions and post-processed uniformly using Adobe Photoshop CS6 (Adobe Systems, Inc., San Jose, CA, USA).

### 2.7. Western Blot Analysis of GFAP Expression

Animals were anesthetized with isoflurane and euthanized by decapitation 24 h after UCCAO surgery. Eyes were quickly removed, and retinas were dissected. Retinal tissue was placed on dry ice and stored at −80 °C. Protein extraction was performed on samples (*n* = 4 per group) by homogenization in ice-cold RIPA buffer with protease and phosphatase inhibitors, following Kvarik et al. [37]. Homogenates were centrifuged at 12,000× g for 10 min at 4 °C, and supernatants were collected for protein analysis. The total protein content was determined using the Bradford assay. Each homogenate was diluted 1:1 with Laemmli buffer and heat-denatured at 95 °C for 5 min. Proteins were resolved by SDS-PAGE, with 25 μL of sample loaded per lane. Following electrophoretic separation, proteins were transferred onto nitrocellulose membranes. Blocking was performed for 5 min at room temperature using EveryBlot Blocking Buffer (BioRad, Hercules, CA, USA) to minimize nonspecific antibody binding. Membranes were incubated overnight at 4 °C with primary antibodies against GFAP (1:1000, G9269, Sigma-Aldrich, Budapest, Hungary) and GAPDH (1:20,000, 2118L, Cell Signaling Technology, Danvers, MA, USA), the latter serving as a housekeeping control. After primary incubation, membranes were washed in Tris-buffered saline containing 0.2% Tween-20 (TBST, pH 7.5) for 30 min. Secondary detection was performed using a goat anti-rabbit IgG (H+L) HRP-conjugated antibody (1:3000, 1721019, BioRad, Hercules, CA, USA), incubated for 2 h at room temperature. Protein bands were visualized using Clarity ECL Western blotting Substrate (BioRad, Hercules, CA, USA). Densitometric quantification of GFAP expression, normalized to GAPDH, was carried out using ImageJ software (version 1.54g).

### 2.8. Apoptosis Signaling Pathway Analysis

Retinas were isolated and stored as described in the previous section. A Mouse Apoptosis Signaling Pathway Array (SARB0074, AssayGenie, Dublin, Ireland) was performed as described by the manufacturer. Retinal tissue (*n* = 4 per group) was homogenized in lysis buffer with protease and phosphatase inhibitors. After 30 min blocking at room temperature, 750 μg of protein from each sample was loaded into the wells and incubated overnight at 4 °C. The membranes were then washed according to the user manual. The detection antibody cocktail was then loaded and incubated overnight at 4 °C followed by washing steps. Before the chemiluminescence detection, HRP-Anti-Rabbit IgG incubation for 2 h at room temperature and washing were performed. Quantification of the protein expressions was performed using ImageJ software (version 1.54g).

### 2.9. Statistical Analysis

The thicknesses of the total retina, as well as the inner, middle, and outer parts, and all individual layers measured by OCT, were standardized as described elsewhere [36,38]. Two-way repeated measures ANOVA and Šídák’s multiple comparisons test were performed to compare the values before the ischemia and 14 days later.

Comparisons of ganglion cell numbers between groups were performed with two-way ANOVA and Tukey’s post hoc test. Results were considered significant if *p* < 0.05.

To evaluate the changes in the expression of the apoptotic factors, signal densities were measured in duplicate after removing the background. The values were then averaged and normalized according to the user manual. Descriptive statistics were performed to see the relative changes in the level of measured signaling molecules. Factors whose relative densities increased by at least 10% after ischemia compared to controls are shown.

Statistical analysis and graphs were produced with GraphPad (9.5) and R Statistical Software (v4.3.2; 2023) using lme4 and ggplot2 packages.

## 3. Results

### 3.1. Thickness of the Retinal Layers

**Figure 1:**
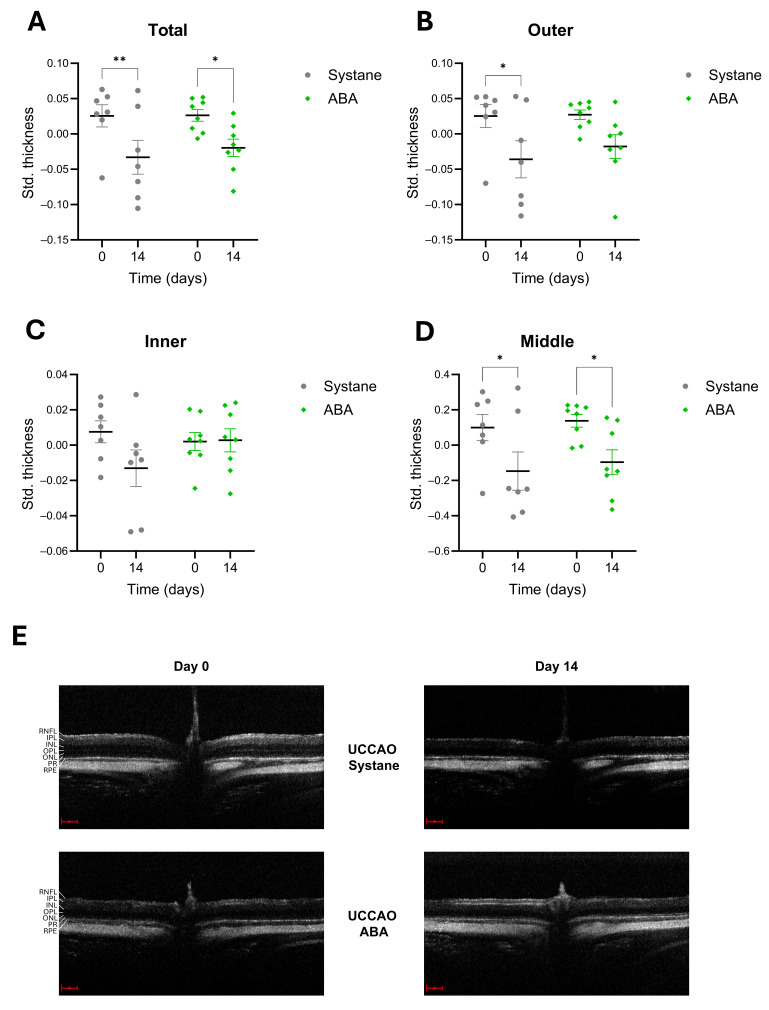
Changes in retinal thickness after ischemia measured by OCT in UCCAO—Systane and UCCAO—ABA groups. The standardized thickness values of the total retina (**A**), the outer (**B**), the inner (**C**) and the middle (**D**) layers are shown at day 0 and day 14. The total retinal thickness decreased significantly in both groups, but not to the same extent (**A**). The outer part of the retina was significantly thinner after UCCAO, but not in the ABA-treated group (**B**). The inner layers did not change significantly after ischemia (**C**), while changes in the middle layers were similar in both groups (**D**). The data are presented as mean ± standard error of mean (SEM). Statistical analysis was performed using two-way ANOVA followed by Šídák’s multiple comparisons. * *p* < 0.05, ** *p* < 0.01 vs. day 0. Representative OCT images. Scale: 100 µm (**E**). Abbreviations: OCT: optical coherence tomography, Std. thickness: standardized thickness, ABA: abscisic acid, UCCAO: unilateral common carotid artery occlusion, RNFL: retinal nerve fiber layer, IPL: inner plexiform layer, INL: inner nuclear layer, OPL: outer plexiform layer, ONL: outer nuclear layer, PR: photoreceptor layer, RPE: retinal pigment epithelium.

**Figure 2:**
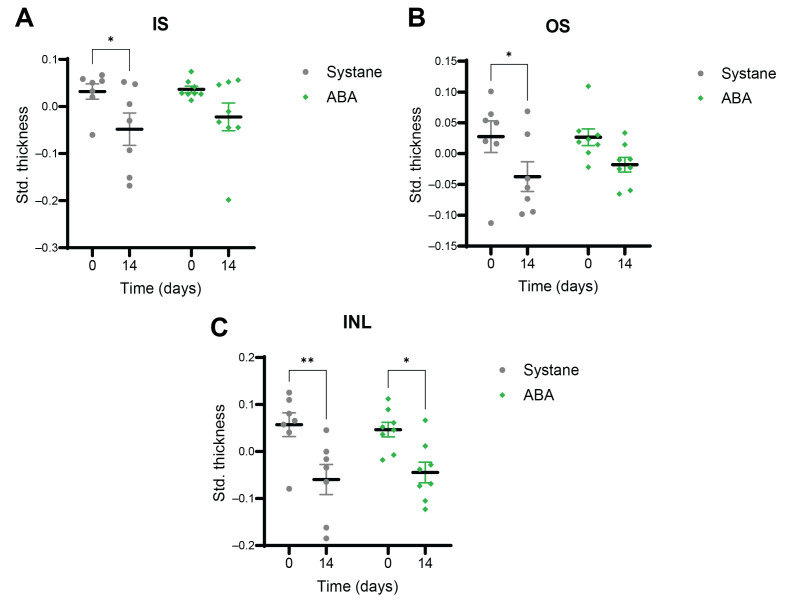
Results of OCT measurements of different retinal layers after ischemia in UCCAO—Systane and UCCAO—ABA groups. The standardized thickness values of the inner (**A**), the outer (**B**) segments of photoreceptors and the inner nuclear layer (**C**) are shown at day 0 and day 14. In the UCCAO—Systane group, significant thinning was observed in all three layers over the 14-day period (**A**–**C**). In comparison, only the INL decreased significantly in the UCCAO—ABA group, but the change was smaller than in the vehicle-treated group (**C**). OS and IS did not decrease significantly in the UCCAO—ABA group during the observational period (**A**,**B**). The data are presented as mean ± standard error of mean (SEM). Statistical analysis was performed using two-way ANOVA followed by Šídák’s multiple comparisons. * *p* < 0.05, ** *p* < 0.01 vs. day 0. Abbreviations: Std. thickness: standardized thickness, INL: inner nuclear layer, IS: inner segments of photoreceptors, OS: outer segments of photoreceptors, ABA: abscisic acid, OCT: optical coherence tomography, UCCAO: unilateral common carotid artery occlusion.

OCT measurements were performed before the surgery and 14 days later in the vehicle-treated (*n* = 7) and in the ABA-treated (*n* = 8) groups (Figure 1 and Figure 2). The main time effect was significant in all illustrated cases; therefore, we could apply post hoc analyses to compare the thicknesses of the layers before and after the hypoxia. Total retinal thickness significantly decreased after UCCAO (*p* = 0.005), but the reduction was less pronounced in the ABA-treated group (*p* = 0.017) (Figure 1A). The thickness of the outer part (photoreceptor layer and pigment epithelial layer) of the retina showed a significant reduction (*p* = 0.014), which was prevented by ABA eye drops (Figure 1B). In contrast, the inner layers (from the nerve fiber layer to the inner nuclear layer) were not as susceptible to ischemia as the outer layers, with no significant thickness changes (Figure 1C). In addition, the change in the thickness of the middle layers (outer plexiform layer and outer nuclear layer) was similar in the two groups (Figure 1D).

After the gross analysis of the retinal structure, each layer was evaluated individually. The photoreceptor layer consists of the inner (IS) and outer (OS) segments of rods and cones. The IS and the OS decreased significantly (*p* = 0.041 and 0.020) in UCCAO, but these damages were prevented by ABA eye drops (Figure 2A,B). The inner nuclear layer (INL) is found between the inner and outer plexiform layers. It contains the cell bodies of bipolar cells, amacrine cells, horizontal cells, Müller glial cells and displaced ganglion cells. The thickness of the INL was reduced after ischemia (*p* = 0.007), while a milder, but still significant change (*p* = 0.021) was observed in the ABA-treated group (Figure 2C).

### 3.2. Photoreceptor Labeling on Retinal Cross Sections

**Figure 3:**
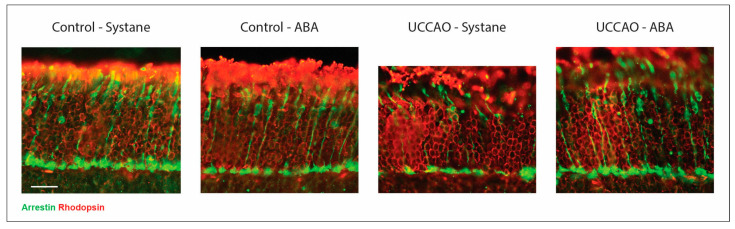
Representative images of photoreceptor labeling. Rods are stained by rhodopsin, while cones are stained by cone arrestin. Scale bar: 20 µm. Abbreviations: ABA: abscisic acid, UCCAO: unilateral common carotid artery occlusion.

Photoreceptor labeling was performed on retinal cross sections for demonstrative purposes. Rods are shown with red and cones with green (Figure 3). Disorganized retinal structure was observed after UCCAO, but it remained similar to controls in the ABA-treated group. The changes in the thickness of the photoreceptor layer were quantified by OCT as described in the previous section. The immunolabeled samples also confirmed those findings (Figure 3).

### 3.3. Retinal Ganglion Cells (Brn3a Labeling)

**Figure 4:**
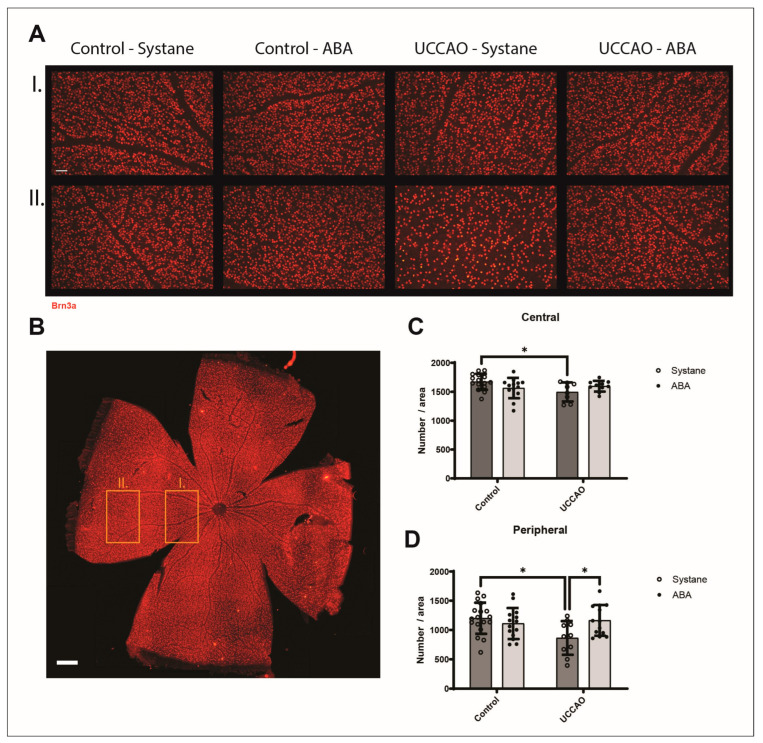
Brn3a labeling of retinal ganglion cells. (**A**) Representative pictures of retinal ganglion cells from the central regions (**I**) and peripheral regions (**II**). Scale bar: 50 µm. (**B**) Representative photo of a whole-mount retina preparation. Rectangle I shows the location of the images of the central region and rectangle II indicates the location of the images of the peripheral region. Scale bar: 500 µm. (**C**) The number of ganglion cells was significantly lower in the UCCAO—Systane group compared to controls, while it was similar to controls in the UCCAO—ABA group in the central region. Area: 0.344 mm^2^. (**D**) Similar findings were observed in the peripheral region as in the central region, but the periphery was more susceptible to ischemia. Area: 0.344 mm^2^. Two-way ANOVA and Tukey’s post hoc test were used as statistical analysis. * *p* < 0.05. Abbreviations: ABA: abscisic acid, UCCAO: unilateral common carotid artery occlusion.

The number of ganglion cells was evaluated in the central and peripheral regions on retinal flat mounts (Figure 4). In the central regions the absolute value of the Brn3a-positive cells was 1672 ± 133 in the Control—Systane, 1565 ± 168 in the Control—ABA, 1497 ± 153 in the UCCAO—Systane and 1596 ± 87 in the UCCAO—ABA groups. In the peripheral regions 1200 ± 259, 1110 ± 254, 864 ± 272, 1164 ± 252 ganglion cells were counted in the Control—Systane, in the Control—ABA, in the UCCAO—Systane and in the UCCAO—ABA groups, respectively. The ganglion cell count decreased significantly after UCCAO (*p* = 0.044), but this degeneration was not observed in the ABA-treated group. Similar to previous findings, the peripheral regions were more sensitive to ischemia. Significant ganglion cell loss occurred 14 days after the surgeries (*p* = 0.013). ABA eye drops prevented this damage, and the number of ganglion cells was significantly higher in the UCCAO—ABA group than in the UCCAO—Systane group (*p* = 0.049).

### 3.4. Retinal Stress (Expression of GFAP)

**Figure 5:**
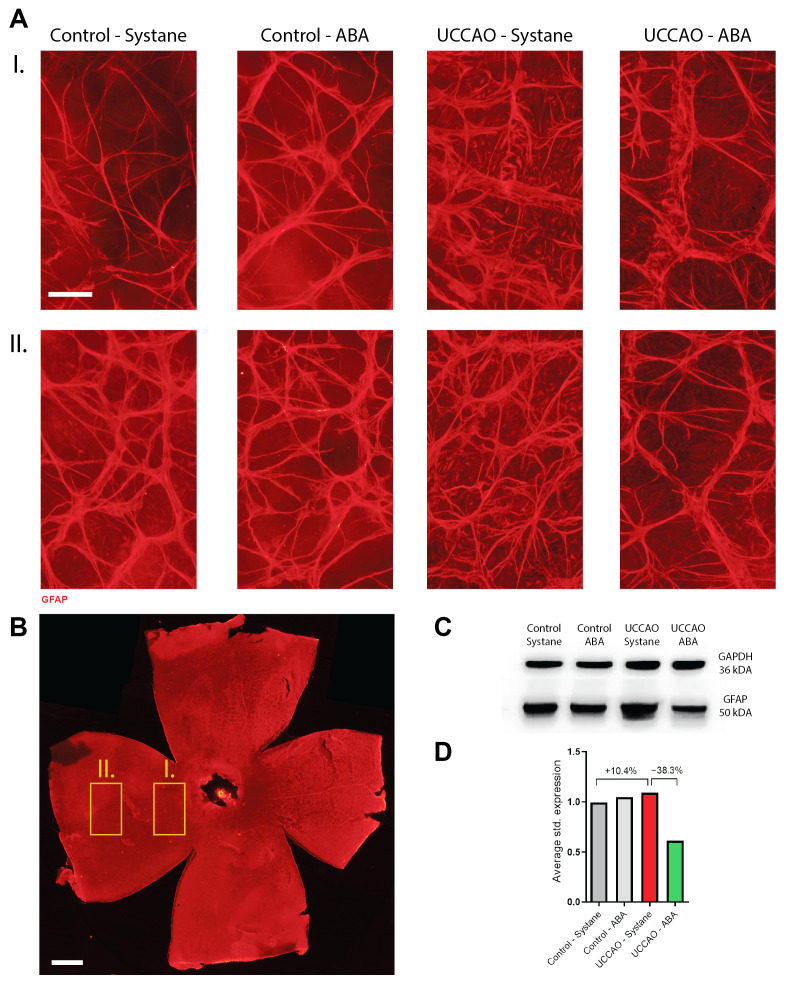
Expression of GFAP in control and UCCAO groups. (**A**) Representative images of GFAP staining on retinal whole-mounts. I: central region. II: peripheral region. Scale bar: 50 µm. (**B**) Representative photo of a whole-mount retina preparation. Rectangle I shows the location of the images of the central region and rectangle II indicates the location of the images of the peripheral region. Scale bar: 500 µm. (**C**) Western blot analysis of GFAP expression. (**D**) Western blot expression of GFAP in the four conditions. Abbreviations: ABA: abscisic acid, UCCAO: unilateral common carotid artery occlusion, GFAP: glial fibrillary acidic protein. GAPDH: glyceraldehyde-3-phosphate dehydrogenase as internal control.

GFAP is a commonly used retinal stress marker, which is overexpressed in reactive Müller glial cells and astrocytes [39]. GFAP expression was visualized in retinal whole-mounts, with images captured from central and peripheral regions (Figure 5A,B). Immunolabeling was performed for demonstrative purposes. The relative GFAP expression was evaluated by Western blot analysis (Figure 5C). Runnings were performed in duplicates (*n* = 4 retinas from individual animals/running). The average fold change was 1.104 after UCCAO compared to controls and the expression was 0.383-fold lower in the ABA-treated group than in the UCCAO—Systane group.

### 3.5. Apoptosis Signaling Pathway Array

**Figure 6:**
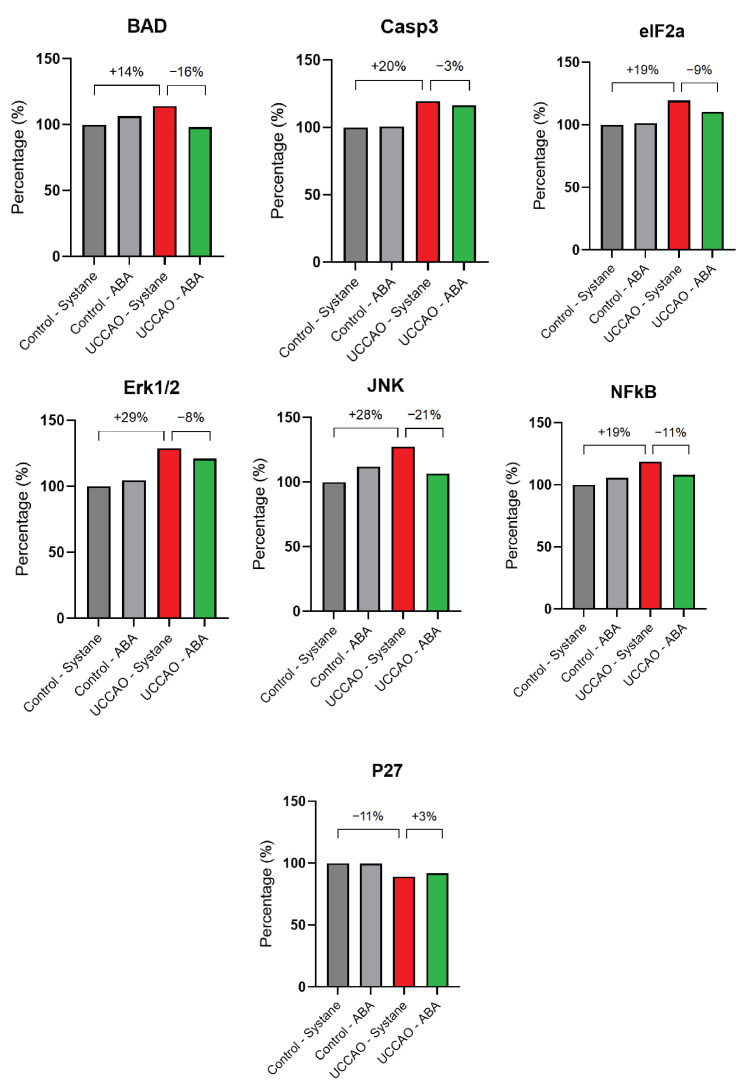
Apoptosis signaling pathway array results. To evaluate the changes in the expression of the apoptotic factors, signal densities were measured in duplicate. The values were then averaged and normalized. Descriptive statistics (relative change in percentages) were performed to see the relative changes in the level of the measured signaling molecules. Abbreviations: BAD: Bcl-2-associated agonist of cell death, Casp3: Caspase-3, eIF2a: eukaryotic translation initiation factor 2 subunit alpha, Erk1/2: extracellular signal-related kinases 1 and 2, JNK: Jun N-terminal kinase, NFkB: Nuclear factor kappa-light-chain-enhancer of activated B cells, P27: cyclin-dependent kinase inhibitor 1B.

Based on the apoptosis signaling pathway array used, the relative expression of the analyzed factors changed in many cases by more than 10% after ischemia (Figure 6). Expression of Bcl-2–associated agonist of cell death (BAD) increased by 14% after ischemia, a change that was prevented by ABA treatment. The relative expression of Caspase-3 (Casp3) was 20% higher after UCCAO, while ABA eye drops decreased this elevation only by 3%. The level of eukaryotic translation initiation factor 2 subunit alpha (eIF2a) increased by 19%, while ABA moderated this elevation by 9%. Extracellular signal-related kinases 1 and 2 (Erk1/2) showed a 29% increase after hypoxic conditions, which was slightly (8%) moderated by administering ABA eye drops. The expression of Jun N-terminal kinase (JNK) was 28% higher after UCCAO, in contrast to the ABA-treated group. Nuclear factor kappa-light-chain-enhancer of activated B cells (NFkB) showed a 19% increase in ischemia, but this change was attenuated by the applied treatment. In addition, the level of cyclin-dependent kinase inhibitor 1B (p27) decreased by 11% after ischemia and a similar level was observed in the ABA-treated group.

## 4. Discussion

In this study, we investigated the potential therapeutic effects of ABA eye drops in ischemic retinopathy. Inadequate oxygen supply is a key factor in the development of the most common non-communicable ocular diseases that lead to visual impairment [9,40,41]. Several disease-specific models are used in experimental practice, but our aim was to describe the general actions of ABA under hypoxic conditions [42,43]. Although a large body of evidence is available about the great therapeutic potential in ABA, only a few articles have been published on its role in retinal diseases [35,44]. We believe that our results can provide a basis for further investigation of the effects of ABA on other specific pathologies.

In our experiments, permanent UCCAO was performed to induce retinal hypoxia in mice. Various forms of this model are used to cause ischemic conditions not only in the brain but also in the retina [36,45,46]. Thickness of the retinal layers was measured by OCT, a non-invasive imaging technique widely used in clinical practice [47]. After analyzing the total retina, the software divided it into inner, middle, and outer regions for further analysis. The inner layers are the nerve fiber, the inner plexiform and the inner nuclear layers. The middle part contains the outer plexiform and the outer nuclear layers, while the outer part includes the photoreceptor and the pigment epithelial layers. The outer layers were particularly prone to ischemia, but ABA eye drops could prevent their degradation. Detailed analysis of photoreceptors showed ischemia-induced damage to both inner and outer segments, but not in the ABA-treated group. Furthermore, rods and cones were visualized with arrestin and rhodopsin immunolabeling, where similar findings were observed. Photoreceptors are essential in proper retinal function, but hypoxia causes their shortening, which impairs function, often irreversibly [48,49,50,51]. Clinical studies have also described that the severity of photoreceptor damage plays a key role in the outcome of ischemia- induced ocular diseases [52,53].

Retinal ganglion cells are essential in visual signal transmission, so their degeneration can easily lead to impaired vision. Ganglion cell loss and its possible prevention have been extensively studied under various hypoxic conditions [54,55,56]. We performed ganglion cell labeling with Brn3a on whole-mount retinas 14 days after the surgeries. Immunohistochemistry was chosen because the SD-OCT devices used in experimental practice are generally not able to independently detect the ganglion cell layer. Calculated ganglion cell complex (GCC) based on OCT measurements can estimate ganglion cell damage, but it is more reliable for a very significant cell loss [57]. Our immunohistochemistry results show a decrease in ganglion cell number after ischemia, which was more pronounced in the peripheral regions of the retina. This model can have a high translational value, because the change in ganglion cell loss was consistent with previously published clinical data. To count the exact ganglion cell loss in patients is technically challenging or almost impossible. To do this, we should know the initial state or evaluate control post-mortem retina samples. However, we can estimate the ganglion cell loss from optical coherence tomography measurements. An observational case-control study shows that patients with diabetic retinopathy had 4.81 µm thinner ganglion cell layer–inner plexiform layer than controls [58]. Another option is to combine standard automated perimetry thresholds and RNFL thickness analyzed with OCT [59]. Medeiros et al. (2013) found that the average ganglion cell loss in glaucoma patients was 28.4%, which is consistent with our findings of an average ganglion cell degeneration of 28% in the peripheral retinal regions [60]. Topical ABA treatment effectively prevented this decrease. The literature suggests that ABA not only preserves the retinal neurons but also has neuroprotective effects in the brain [61].

To explore underlying mechanisms, apoptosis array analysis was performed to assess signaling pathways modulated by ABA under hypoxia. Spinelli and coworkers described that hypoxia stimulates ABA release followed by increased nitrogen monoxide (NO) production in rat cardiomyocyte H9c2 cells [62]. It is also known that ABA exerts its functions through Lanthionine synthetase C-like protein 1 and 2 (LANC-1 and LANCL-2) receptors [44]. Silencing these receptors leads to functional decline in rat cardiomyocytes, while their overexpression reduces the damage caused by hypoxia and reoxygenation [62]. Inflammatory processes also contribute to functional and morphological changes after hypoxia [63]. NFkB is a key inflammatory mediator; it regulates different aspects of the innate and the adaptive immune systems [64]. Inhibition of NFkB-related pathways, such as NFkB/NLRP3/IL-18 can help to protect the retina after LPS-induced inflammation [65]. Another study showed that ABA suppresses NFkB activity, which is most probably a peroxisome proliferator-activated receptor gamma (PPAR γ)-dependent mechanism [66]. Consistent with previous reports, we observed increased NFκB expression after ischemia, which was attenuated by ABA treatment.

BAD is a member of the Bcl-2 family with proapoptotic actions. It promotes cell death when it is unphosphorylated [67]. However, increased BAD phosphorylation can occur under ischemic conditions as a temporary compensatory mechanism during activation of the mitogen-activated protein kinase (MAPK) pathways. This reaction can be a stress-induced survival signal [68,69,70]. In our study, ischemia caused phospho-BAD (p-BAD) elevation but not after administering ABA eye drops. It has been known for more than 20 years that BAD is cleaved by Casp3, a process that enhances the proapoptotic property of BAD [71]. In addition, BAD-BAX-Casp3 cascade has a role in the regulation of synaptic vesicle pools [72]. Interestingly, ABA had different effects on BAD and Casp3 in retinal tissue. The level of Casp3 was elevated after oxygen deprivation and ABA could not attenuate this response. JNKs are part of the MAP-kinases and have a key role in extrinsic and intrinsic mitochondrial apoptotic pathways [73]. The expression of JNK increased almost 30% after retinal ischemia, but it remained similar to controls after ABA treatment. ERK1/2, also a key member of the MAPK pathway, can promote or inhibit the apoptotic processes depending on the stimuli, cell type and the duration of its activation [74]. Our results suggest that ABA does not markedly affect the level of ERK1/2. eIF2a downregulates protein synthesis when serine 51 is phosphorylated [75]. P27, a cyclin-dependent kinase involved in apoptosis and cell-cycle regulation, was slightly elevated after ischemia [76]. Furthermore, a study has shown that ABA can promote quiescence via LANCL2- and PPARγ-induced MAPK activation in prostate cancer [77]. Based on the literature and our results, ABA regulates the apoptotic pathways under hypoxic conditions, but further research is needed to clarify the underlying signaling mechanisms. One limitation of the present study is that statistical evaluation of the apoptosis array results was not carried out, since this would have required a considerably larger number of animals than was ethically feasible. Furthermore, we did not investigate how apoptotic markers vary across different time points. For this reason, the data presented here should be interpreted as a descriptive snapshot of molecular alterations at 24 h after ischemia, rather than a comprehensive temporal analysis.

In addition to the previously described therapeutic potential, ABA can also be used as a biomarker in hypoxic diseases. A study showed that patients with asthma, acute respiratory distress syndrome (ARDS) or chronic obstructive pulmonary disease (COPD) have significantly lower serum ABA levels than healthy controls [78,79]. ABA may also show the disease progression in glioma patients, because its level was lower in high-grade gliomas than in low-grade stages [80].

In summary, our results show that ABA has a protective role in ischemic retinopathy and may complement the available treatment options in the future. A huge advantage is that ABA is completely natural, it can be found in fruits and vegetables [81]. Furthermore, unlike most therapeutic options available for ischemic retinopathy, in our study it was administered non-invasively at a concentration of 50 µM. This concentration was chosen because in 2018, Chaqour and coworkers found that intravitreal injection of 50 µM ABA reduced the number of Tunel-positive cells, slowed down the spread of the superficial capillary plexus to the retinal edge and inhibited endothelial cell sprouting in a mouse model of retinopathy of prematurity. In conclusion, ABA in 50 µM concentration inhibited neovascularization without affecting cell viability [35]. The therapeutic applicability of ABA is also underlined by the fact that a clinical trial has shown that it has no side effects [82]. However, more research is needed to analyze its long-term efficacy, safety and translational potential in eye-related conditions. Our study may provide a basis for further investigation of the effects of ABA in different vision-threatening retinal pathologies.

## Data Availability

The original contributions presented in the study are included in the article; further inquiries can be directed to the corresponding author.

## Abbreviations

The following abbreviations are used in this manuscript:

ABA	Abscisic acid
ADHD	Social interaction in attention-deficit/hyperactivity disorder
ARDS	Acute respiratory distress syndrome
BAD	Bcl-2-associated death promoter
BAX	Bcl-2-associated X protein
Brn3a	Brain-specific homeobox/POU domain protein 3A
BSA	Bovine serum albumin
Casp3	Caspase-3
COPD	Chronic obstructive pulmonary disease
DMSO	Dimethyl sulfoxide
eIF2a	Eukaryotic translation initiation factor 2 subunit alpha
Erk1/2	Extracellular signal-related kinases 1 and 2
GAPDH	Glyceraldehyde-3-phosphate dehydrogenase
GCC	Ganglion cell complex
GFAP	Glial fibrillary acidic protein
HRP	Horseradish peroxidase
IL-18	Interleukin-18
INL	Inner nuclear layer
IS	Inner segments of photoreceptors
JNK	Jun N-terminal kinase
LANC-1, LANCL-2	Lanthionine synthetase C-like protein 1 and 2
MAPK	Mitogen-activated protein kinase
NDS	Normal donkey serum
NFkB	Nuclear factor kappa-light-chain-enhancer of activated B cells
NLRP3	NLR family pyrin domain containing 3
NO	Nitrogen monoxide
NRF2	Nuclear factor erythroid 2-related factor 2
OCT	Optical coherence tomography
OS	Outer segments of photoreceptors
p27	Cyclin-dependent kinase inhibitor 1B
p-BAD	Phospho-BAD
PBS	Phosphate-buffered saline
PFA	Paraformaldehyde
PPAR γ	Peroxisome proliferator-activated receptor gamma
RIPA	Radioimmunoprecipitation assay
Std. thickness	Standardized thickness
SD-OCT	Spectral domain optical coherence tomography
SEM	Standard error of mean
UCCAO	Unilateral common carotid artery occlusion
VEGF	Vascular endothelial growth factor
